# Postpartum modern family planning among women living with HIV attending care at health facilities in Busia County, Kenya

**DOI:** 10.1186/s40834-024-00319-2

**Published:** 2024-11-12

**Authors:** Florence Zawedde Tebagalika, Derrick Kimuli, Dennis Walusimbi, Edna Nyang’echi, Louisa Ndunyu

**Affiliations:** 1 School of Public Health and Community Development, Department of Public Health, Maseno University, Kisumu, Kenya; 2 Social & Scientific Systems, Inc., a DLH Holdings Company, Kampala, Uganda; 3 Galen Health Consulting Group, Kampala, Uganda

**Keywords:** Modern FP, Women living with HIV, Postpartum family planning, Kenya

## Abstract

**Background:**

For women living with the human immunodeficiency virus (WLHIV), preventing untimed pregnancies during the postpartum period reduces vertical transmission and improves other maternal and child health outcomes. In Kenya, Busia County’s HIV prevalence and mother-to-child transmission rate are higher than the national average yet uptake of postpartum family planning (PPFP) is generally low. This study examined health system factors influencing the consistent use of PP modern FP methods among WLHIV in Busia County.

**Methods:**

A retrospective study involving 314 WLHIV with children aged 12–24 months who were chosen using systematic random sampling was conducted from February to March 2024 from outpatient clinics in Busia County. Additionally, 14 health providers were purposively sampled as key informants. Quantitative data was collected using a pretested questionnaire, while qualitative data was gathered through key informant interview guides. Quantitative data was analyzed using STATA 15 with descriptive statistics, logistic regression, and Chi-square tests, while a deductive thematic analysis was used for qualitative data.

**Results:**

The mean age of the participants was 32.06 (± 6.00) with the majority (51.27) aged between 25 and 34 years, married (74.84%) and unemployed (77.39%). Overall, 73.25% had used postpartum (PP) modern family planning (FP) methods, but only 52.55% reported consistent use throughout the first year postpartum. The only factors found to increase the odds of PPFP use were being married (aOR 3.34, 95% CI 1.58–7.07, *p* = 0.002), being escorted by a preferred person during seeking maternal and child health services (aOR 2.29, 95% CI 1.36–3.83, *p* = 0.002), and perceiving that they were provided information on all types of FP (aOR 2.33, 95% CI 1.19–4.16, *p* = 0.012). Persistent stock-outs and inadequate counseling hindered consistent PPFP use.

**Conclusion:**

The study identified gaps in the consistent use of PP modern FP methods among WLHIV in Busia County, influenced by the availability of FP information and health system factors. Addressing stock-outs and improving counseling during clinic visits and pregnancy are crucial for improving FP service delivery and reducing maternal and child health risks in high HIV-incidence areas like Busia County.

**Supplementary Information:**

The online version contains supplementary material available at 10.1186/s40834-024-00319-2.

## Background

Modern family planning includes the use of hormonal methods, condoms, and voluntary sterilization to space or limit births [1]. Postpartum Family Planning (PPFP) prevents unintended or closely spaced pregnancies within the first year after childbirth and can extend up to two years [1]. PPFP as a component of general family planning (FP) is widely accepted as a life-saving intervention for mothers and their children [1]. The rationale is that the majority of women in the postpartum period have an unmet need for FP [2]. PPFP using modern methods is essential for the prevention of maternal and child deaths [3]. Maternal and child health outcomes are poorest when pregnancies are closely spaced pregnancies (i.e. within the first year postpartum) during which time there is the highest risk for adverse outcomes [4]. Studies have also shown that the risk of child mortality is highest at birth intervals of less than 12 months, decreasing by 13% and 25% at birth intervals of 24 and 36 months respectively [5]. In developing countries, this risk is further exacerbated by the high risk of mother-to-child transmission (MTCT) of the human immunodeficiency virus (HIV) making PPFP a priority intervention among mothers/women living with HIV (M/WLHIV) [6].

In sub-Saharan Africa (SSA), women between the age of 15–49 years bear the brunt of HIV where they account for about 60% of the total number of people living with HIV [7]. The women are also at risk of unintended pregnancies but more importantly, at risk of shortly timed birth intervals (< 12 months) [6]. The postpartum period is a time of increased biological susceptibility to pregnancy-related sepsis. Enabling WLHIV to avoid unintended pregnancies during the postpartum period can reduce vertical transmission and maternal mortality associated with HIV infection. PPFP is a cost-effective strategy for the prevention of mother-to-child transmission (PMTCT) and the reduction of maternal/infant morbidity and mortality [6]. However, rather than the provision of FP being a critical component in PMTCT, it has been observed that the two remain estranged [8]. Therefore, this has resulted in low but persistent above-target MTCT rates observed in SSA countries despite higher levels of antiretroviral therapy (ART) coverage [9] contributing to the status of the epidemic in the countries.

In Kenya, similar to what is observed in SSA [7], HIV disproportionately affects women with a prevalence of 6.6% which is more than twice that in men [10]. In the country, 97.3% of pregnant women living with HIV attend at least one antenatal care visit, with an MTCT rate of 6.7 [9]. Unintended pregnancy is a public health issue in Kenya, 14% of married and 19% of unmarried women face an unmet need for FP [11]. In Busia County, 78% of pregnancies among WLHIV are unplanned, and modern FP use is only 56%. The County’s fertility rate (3.7) exceeds both the national average (3.4) and the 2030 target (2.6), partly due to a high unmet need for FP (19% vs. 14% nationally). Limited information on PPFP, high fertility, high unmet need, and high HIV incidence (6.7%) contribute to an elevated MTCT rate of 9.7%, hindering progress toward national and global targets [6]. PPFP could be one of the missing links in narrowing the MTCT rate to less than what is currently observed in the county [10]. This study explored the uptake and health system factors that influenced the uptake of PPFP among WLHIV as a recommended prong in PMTCT (preventing unintended pregnancies among WLHIV).

## Methods

### Study design, setting and population

The study was a retrospective study conducted during February and March 2024 at 7 health facilities in Busia County (Busia County referral hospital, Alupe Sub-county Hospital, Teso North Sub-county Hospital, Khunyangu Sub-county Hospital, Nambale Sub-county Hospital, Port Victoria Sub-county Hospital and Samia Sub-county Hospital). These facilities were chosen because they not only provided the adequate sample size needed for the study but also represented the primary facilities providing ART to most WLHIV in Busia County. The study population consisted of WLHIV of reproductive age (15–49 years) who were seeking HIV care at the facilities and had biological children between 12 and 24 months old. Health workers involved in HIV and/or maternal and childcare services provision at the selected facilities were also invited to participate in the study.

### Sampling

The study sample size was calculated using the adjusted Fisher’s formula for small populations [12, 13]. With an initial sample size of 372, a population size of 1,185, and a 10% non-response rate, the final sample size was 314. Proportionate sampling was applied across health facilities, followed by systematic random sampling to establish the sampling interval based on records of WLHIV receiving ART care at 7 Sub County hospitals. Purposive sampling was used to select 14 health workers involved in HIV and MCH services for qualitative data collection [14].

### Study variables and measurements

The dependent variable was PPFP uptake denoted as a Yes or No and defined as the use of a modern FP method *consistently* during the 12 months after the birth of the child. The independent variables were the client-perceived health system factors (FP counselling, PPFP counselling, adequate information on PPFP, payment for PPFP, waiting time, availability of services/FP commodities, distance to the health facility, health facility operation hours, PPFP safety concerns, confidentiality), Health provider- related health system factors (adequate and appropriate staffing, adequate support supervision, staff trained & mentorship, staff motivation of health workers, availability of variety contraceptives and stock-outs of FP commodities).

### Statistical analysis

The data collected using the interviewer-administered questionnaire were downloaded in MS-Excel, where preliminary cleaning was performed. The data was then exported into STATA 15 [15] for further cleaning and analysis. Descriptive analysis was conducted for discrete (e.g., age) and categorical variables (e.g., marital status) using frequencies and percentages. Numerical data were summarized with means and standard deviations for normal distributions, and medians with interquartile ranges for skewed data. Chi-square or Fisher’s exact test assessed differences in PPFP use and categorical variables. Multivariate analysis used logistic regression to calculate unadjusted and adjusted odds ratios at 5% significance. Model assumptions were verified before finalizing. Qualitative data collected using a key informant guide from 14 healthcare workers were transcribed and analyzed using thematic analysis [16], based on deductive themes developed from the WHO health systems building blocks framework [17].

## Results

### Characteristics of the study participants

A total of 314 women participated in the study, with an average age of 32.06 years (standard deviation [SD] ± 6.00). The age distribution among the participants followed a normal pattern (Shapiro-Wilk Test, *p* = 0.695), spanning from 19 to 45 years. Notably, the largest proportion fell within the 24–34 age bracket (161, 51.27%). Additionally, a majority (177, 56.37%), reported primary education as their highest level of attainment unemployed (243, 77.39%), married (235, 74.84%), and (194, 61.78%) had received HIV care for at least 5 years or more. Furthermore, most participants had fewer than 5 pregnancies (222, 70.70%), their last pregnancy occurred more than 2 years ago (250, 79.62%), they did not plan their current pregnancy (149, 58.20%), and had a history of contraceptive use before the postpartum period (240, 76.43%). Moreover, statistically significant differences in PPFP use were observed based on marital status (*p* = 0.001), duration since the last pregnancy (*p* = 0.035), and history of contraceptive use before the postpartum period (*p* = 0.004). The detailed characteristics of the participants are presented in Table 1.

**Table 1:** Participant characteristics and bivariate analysis of participant characteristics

Variable	Category	PPFP Use		Total (314)	*P* value
		No (149)	Yes (165)		
Age group	15–24	15 (40.55)	22 (59.46)	37 (11.78)	0.239
	25–34	72 (44.73)	89 (55.28)	161 (51.27)	
	35–45	62 (53.45)	54 (46.56)	116 (36.94)	
Highest level of education	None	11 (40.75)	16 (59.26)	27 (8.60)	0.254
	Primary	79 (44.64)	98 (55.37)	177 (56.37)	
	≥Secondary	59 (53.64)	51 (46.37)	110 (35.03)	
Employment status	Unemployed	113 (46.51)	130 (53.5)	243 (77.39)	0.533
	Employed	36 (50.71)	35 (49.3)	71 (22.61)	
Marital status	Single-non regular	37 (71.16)	15 (28.85)	52 (16.56)	0.001**
	Single regular	12 (60)	8 (40)	20 (6.37)	
	Married	97 (41.28)	138 (58.73)	235 (74.84)	
	Living together	3 (42.86)	4 (57.15)	7 (2.23)	
Duration in HIV care	< 5 years	5 (7.05)	66 (92.96)	120 (38.22)	0.494
	5 or more years	95 (48.97)	99 (51.04)	194 (61.78)	
Gravidity	< 5	105 (47.3)	117 (52.71)	222 (70.70)	0.932
	≥ 5	44 (47.83)	48 (52.18)	92 (29.30)	
When was last pregnancy	< Year	3 (100)	0 (0)	3 (0.96)	0.035*
	1–2 years	9 (34.62)	17 (65.39)	26 (8.28)	
	2 years	115 (46)	135 (54)	250 (79.62)	
	first child	22 (62.86)	13 (37.15)	35 (11.15)	
Planned to have child	No	59 (39.6)	90 (60.41)	149 (58.20)	0.11
	Yes	32 (29.91)	75 (70.1)	107 (41.80)	
History of modern contraceptive use before the postpartum period	No	46 (62.17)	28 (37.84)	74 (23.57)	0.004**
	Yes	103 (42.92)	137 (57.09)	240 (76.43)	

Note significant codes at *p < 0.05 and **p < 0.01

### Uptake of modern PPFP among WLHIV in Busia County, Kenya

Overall, 73.25% (95% Confidence Interval (CI) 68.00–78.07) of the participants had utilized PPFP at some point. However, only 52.55% (95% CI 46.86–58.18) reported consistent PPFP usage throughout the first year postpartum. The utilization of PPFP by contraceptive method revealed the following distribution: Sterilization (3, 1.82%), IUD (1, 0.61%), Orals (3, 1.82%), Injectables (53, 32.12%), Implants (90, 54.55%), FAM (2, 1.21%), and Condoms (10, 6.06%). Comparatively, injectables and implants were the most utilized methods. Utilization of PPFP at different time points post-delivery showed the following distribution: Immediately: (7, 4.27%), At six weeks (62, 37.80%), and more than 6 weeks (95, 57.93%).

### Relationship between client-centred service factors and uptake of modern PPFP

Table 2 below presents detailed study findings regarding the relationship between client-centred service factors and the uptake of modern PPFP. Overall, the findings indicated that PPFP users were more likely to have utilized/received a package of maternal health services (antenatal care [ANC], maternity [MAT], and postnatal care [PNC]) (161, 52.45%) at a health facility, were accompanied by a preferred partner during health service visits (79, 64.76%), attended some Maternal and Child Health (MCH) services at the facility where they received ART (160, 53.16%), not been counseled for FP during a clinic visit (24, 58.54%), counseled on PPFP during the pregnancy (142, 54.62%), provided with adequate information on PPFP (140, 54.69%), informed about all available PPFP methods (139, 58.16%), aware of the availability of PPFP methods at their facility of care (164, 53.08%), informed that modern PPFP methods at the facility were free (156, 54.17%) and not asked to pay for PPFP methods (52, 55.92%).

Moreover, the findings also indicated that PPFP users were more likely to feel that the waiting time was acceptable (135, 56.02%), perceived health workers as receptive (159, 53.18%), ever encountered unavailability of the preferred PPFP method (81, 61.84%), traveled more than 5 km to the facility they sought health care (100, 53.77%), did not find health facility operational hours adequate for their needs (164, 52.91%), were confident in the efficacy of modern PPFP methods (154, 55%), lacked confidence in the health facility’s ability to store their records (163,52.59%), were not satisfied with health workers’ service provision ability (164, 53.43%), and did not perceived health workers as competent (159, 52.65)%). However, significant differences (*p* < 0.05) were only observed in the following variables: being escorted by a preferred person during MCH service seeking, perceiving that they were provided information on all types of FP, finding the wait time at the facility acceptable, encountering the need for a specific type of PPFP method that was unavailable, and feeling confident in the efficacy of modern contraceptives to prevent pregnancy.

**Table 2:** Bivariate analysis of client-centred service factors and PPFP uptake

Variable	Category	PPFP Use		Total (314)	*P* value
		No (149)	Yes (165)		
MCH Services	None	1 (50)	1 (50)	2 (0.64)	0.821
	ANC Only	0 (0)	1 (100)	1 (0.32)	
	ANC & Mat Only	2 (50)	2 (50)	4 (1.27)	
	ANC, MAT & PNC	146 (47.56)	161 (52.45)	307 (97.77)	
Escorted by preferred person during MCH service seeking	No	105 (55.27)	85 (44.74)	190 (60.90)	0.001**
	Yes	43 (35.25)	79 (64.76)	122 (39.10)	
Attended some of the MCH services at the ART facility	No	5 (71.43)	2 (28.58)	7 (2.27)	0.198
	Yes	141 (46.85)	160 (53.16)	301 (97.73)	
Ever been counselled on FP during any clinic visit	No	17 (41.47)	24 (58.54)	41 (13.06)	0.41
	Yes	132 (48.36)	141 (51.65)	273 (86.94)	
Taught or counselled on FP during the pregnancy	No	31 (57.41)	23 (42.6)	54 (17.20)	0.107
	Yes	118 (45.39)	142 (54.62)	260 (82.80)	
Perceived that they were given enough information	No	33 (56.9)	25 (43.11)	58 (18.47)	0.111
	Yes	116 (45.32)	140 (54.69)	256 (81.53)	
Perceived that they were given information on all types of FP	No	49 (65.34)	26 (34.67)	75 (23.89)	< 0.001***
	Yes	100 (41.85)	139 (58.16)	239 (76.11)	
Were aware of the availability of modern FP methods at the facility	No	4 (80)	1 (20)	5 (1.59)	0.142
	Yes	145 (46.93)	164 (53.08)	309 (98.41)	
Were aware that modern FP methods at the facility were FREE	No	13 (61.91)	8 (38.1)	21 (6.80)	0.154
	Yes	132 (45.84)	156 (54.17)	288 (93.20)	
Had ever been asked to pay for PPF	No	108 (48.87)	113 (51.14)	221 (70.38)	0.438
	Yes	41 (44.09)	52 (55.92)	93 (29.62)	
Perceived the wait time at the facility acceptable	No	43 (58.91)	30 (41.1)	73 (23.25)	0.025*
	Yes	106 (43.99)	135 (56.02)	241 (76.75)	
Perceived health workers at the facility as receptive to them	No	8 (72.73)	3 (27.28)	11 (3.55)	0.091
	Yes	140 (46.83)	159 (53.18)	299 (96.45)	
Always received care and medicines they needed at the facility	No	40 (41.67)	56 (58.34)	96 (30.57)	0.173
	Yes	109 (50)	109 (50)	218 (69.43)	
Had ever needed type of PPFP and it was not available	No	99 (54.1)	84 (45.91)	183 (58.28)	0.005**
	Yes	50 (38.17)	81 (61.84)	131 (41.72)	
Distance of health facility from home	< 5 Km	63 (49.22)	65 (50.79)	128 (40.76)	0.603
	≥ 5 Km	86 (46.24)	100 (53.77)	186 (59.24)	
Perceived facility operational hours adequate for their needs	No	146 (47.1)	164 (52.91)	310 (98.73)	0.267
	Yes	3 (75)	1 (25)	4 (1.27)	
Were confident in the ability of modern contraceptives to prevent a pregnancy	No	23 (67.65)	11 (32.36)	34 (10.83)	0.013*
	Yes	126 (45)	154 (55)	280 (89.17)	
Were confident in the ability of records to be kept safety at the health facility	No	147 (47.42)	163 (52.59)	310 (98.73)	0.918
	Yes	2 (50)	2 (50)	4 (1.27)	
Were satisfied with the health provider regarding services provided	No	143 (46.58)	164 (53.43)	307 (97.77)	0.056
	Yes	6 (85.72)	1 (14.29)	7 (2.23)	
Felt like the heath worker was competent	No	143 (47.36)	159 (52.65)	302 (96.18)	0.857
	Yes	6 (50)	6 (50)	12 (3.82)	

Note significant codes at *p < 0.05, **p < 0.01, ***p < 0.001

### Client-centered service factors associated with the uptake of modern PPFP

Table 3 presents client-perceived health system factors associated with modern PPFP uptake. In the unadjusted analysis, PPFP uptake was more prevalent among WLHIV who were married compared to those who were single with a non-regular partner (uOR 3.51, 95% CI 1.83–6.75, *p* < 0.001), those whose last pregnancy occurred 1–2 years preceding the study compared to within 1 year (uOR 3.20, 95% CI 1.11–9.22, *p* = 0.032), had a history of contraceptive use compared to none (uOR 2.19, 95% CI 1.28–3.73, *p* = 0.004), were escorted by a preferred person during seeking MCH services compared to not (uOR 2.27, 95% CI 1.42–3.63, *p* = 0.001), perceived that they were provided information on all types of FP compared to not (uOR 2.62, 95% CI 1.53–4.50, *p* < 0.001), perceived the facility wait time as acceptable compared to those who did not (uOR 1.83, 95% CI 1.07–3.10, *p* = 0.026), felt confident in the ability of modern FP methods to prevent pregnancy compared to those who did not (uOR 2.56, 95% CI 1.20–5.44, *p* = 0.015), and those who had ever encountered the need for a specific type of PPFP method that was unavailable (uOR 1.91, 95% CI 1.21–3.02, *p* = 0.006).

In the adjusted analysis, the only factors found to increase the odds of PPFP use were being married (aOR 3.34, 95% CI 1.58–7.07, *p* = 0.002), being escorted by a preferred person during seeking MCH services (aOR 2.29, 95% CI 1.36–3.83, *p* = 0.002), and perceiving that they were provided information on all types of FP (aOR 2.33, 95% CI 1.19–4.16, *p* = 0.012).

**Table 3:** Client-centered service factors associated with PPFP use

Variable	Category	uOR	*p* value	aOR	*p* value
Marital status	Single-non regular	1 (Reference)		1 (Reference)	
	Single regular	1.64 (0.56–4.83)	0.365	2.37 (0.72–7.84)	0.157
	Married	3.51 (1.83–6.75)	< 0.001	3.34 (1.58–7.07)	0.002**
	Living together	3.29 (0.66–16.45)	0.148	5.20 (0.79–33.96)	0.085
When was last pregnancy	< Year	1 (Reference)		1 (Reference)	
	1–2 years	3.20 (1.11–9.22)	0.032	1.81 (0.53–6.30)	0.345
	2 years	1.99 (0.96–4.11)	0.065	1.14 (0.46–2.83)	0.773
	first child	1 (omitted)			
History of modern contraceptive use	No	1 (Reference)		1 (Reference)	
	Yes	2.19 (1.28–3.73)	0.004	1.56 (0.83–2.94)	0.172
Escorted by preferred person during MCH service seeking	No	1 (Reference)		1 (Reference)	
	Yes	2.27 (1.42–3.63)	0.001	2.29 (1.36–3.83)	0.002**
Perceived that they were given information on all types of FP	No	1 (Reference)		1 (Reference)	
	Yes	2.62 (1.53–4.50)	< 0.001	2.33 (1.19–4.16)	0.012*
Perceived the wait time at the facility acceptable	No	1 (Reference)		1 (Reference)	
	Yes	1.83 (1.07–3.10)	0.026	1.46 (0.78–2.73)	0.231
Were confident in the ability of modern contraceptives to prevent a pregnancy	No	1 (Reference)		1 (Reference)	
	Yes	2.56 (1.20–5.44)	0.015	1.87 (0.80–4.37)	0.147
Had ever needed type of PPFP and it was not available	No	1 (Reference)		1 (Reference)	
	Yes	1.91 (1.21–3.02)	0.006	1.48 (0.88–2.48)	0.135

Note significant codes at *p < 0.05, **p < 0.01

### Health service-related factors influencing the uptake of modern PPFP uptake among WLHIV in Busia County Kenya

To assess health provider-related factors influencing the uptake of modern PPFP among WLHIV in Busia County, Kenya, the study qualitatively assessed aspects regarding staffing levels, training, and motivation, alongside the critical role of support supervision and stewardship on PPFP service integration. The findings are sub-themed below in reference to the Adapted from the WHO health system building framework [17].

### Theme: health care workforce

#### Sub-theme 1: staffing levels

The findings of the KII guide revealed significant concerns about staffing levels, training, and mentorship. There is a clear indication of insufficient staffing levels across the facilities, with many respondents highlighting the inadequacy of the workforce to meet the demand for PPFP services among WLHIV. Additionally, the lack of comprehensive training, especially in newer or specific guidelines on PPFP methods, affects service quality and availability.

*“this facility they only two… They’re not [adequate]… they are understaffed…We require like five[staff].” -****KII- 2***

*“We’re running on a skeleton crew*… *and not all are up-to-date on the latest PPFP guidelines.” -****KII- 15***

*“Okay we can’t be enough okay as per now… Atone point in time as an officer*,* you supposed to have some break*.*” -****KII 3***

*“In MCH we are three… there is a shortagebecause sometimes we have workloads higher than what we can manage… out of the 3 only 1 has received formal training.” –****KII- 4***

#### Sub-theme 2: staffing training

The interviews revealed that staff training was a crucial but often inadequately addressed factor impacting the uptake of modern PPFP among WLHIV in Busia County. Training gaps, both in frequency and coverage, limit healthcare workers’ ability to offer updated and comprehensive PPFP services. Moreover, the need for continuous professional development and training in PPFP guidelines and practices was highlighted as essential for enhancing service delivery and ensuring healthcare workers are equipped with the necessary skills and knowledge to meet the diverse needs of WLHIV.

“*In terms of staff*,* we are only two… not really sufficient…I think I am the only one who went for a refresher course last year.” –****KII- 1***

*“it is integrated it’s in one room…Am not aware [of specific policies or SOPs]*.*” –****KII- 2***

*“In MCH we are three… there is a shortage because sometimes we have workloads higher than what we can manage…out of the 3 only 1 has received formal training.” –****KII- 4***

*“Nurses in MCHare not trainedto offer permanent FP methods… but they are doing referrals.” –****KII- 8***

#### Sub-theme 3: support supervision

The study findings also indicated the critical aspect of support supervision as a component in enhancing PPFP service delivery among WLHIV in Busia County. In this regard, the findings showed the lack of regular, structured visits by Senior Health Officials to provide on-site guidance, review service delivery practices, and address challenges faced by staff. The infrequency and general focus of support supervision visits were inadequate for addressing the specific needs and challenges of PPFP service delivery.

*“The county or sub-county health management team… they just come forgeneral supervision*,* maybe MCH supervision*, *and it’s quarterly.” -****KII-1***

*“No county comesI don’t know they come after every three months.” -****KII- 2***

*“Okay supervisions we have had quite a number especially the HMT they usually come…Especially in documentation*. *They have helped us.” - –****KII-3***

#### Sub-theme 4: health information

The use and management of health information varied, with some facilities attempting regular data reviews to improve service delivery. However, specific data targeting PPFP uptake among WLHIV was not consistently analyzed or utilized for decision-making. Therefore, data specific to PPFP among WLHIV is not being distinctively reviewed or utilized for targeted improvements, indicating a gap in strategic data management practices.

*“For now*,* none… we just work as the norm.There’s no that*,* that thing for checking for PPFP data for WLHIV*.*“****- KII-1***

*“they review them but not specifically for people living with HIV*… *it’s every end of the month the first week of every month.” -****KII- 2***

*“We haven’t really looked into PPFP data separately*… *it’s usually lumped with general family planning data.“****- KII-15***

*“That I have not attended any [facility performance data reviews]… maybe Salome has attended; she’s the in charge****- KII- 3***

#### Sub-theme 5: staff motivation

Motivation among staff appeared to be driven primarily by personal commitment and the achievement of targets. Despite not being directly quoted on motivation, the implication of personal commitment and target achievement in several responses suggests that staff are primarily motivated by intrinsic factors and the desire to meet specific health outcomes.

*“For me*,* it’sabout personal commitmentand also we have targets to achieve… so we push ourselves towards that.” -****KII- 1***

*“Three…Yes… To enable that spacing in between their children*.*“****- KII- 13***

*13*,* indicating a motivation driven by the goal of ensuring child spacing.*

*“I think I alsohave targets to achieve.“****- KII- 8***

*“So a client who comes and wants BTL. The permanent method. We have*,* us nurses we have not been trained in that so we just refer them to the clinic to see the gynecologist then they arebooked to be seen for an appointment.”****KII- 6***

### Theme: commodity-related factors

Regarding the assessment of commodity-related factors influencing the uptake of modern PPFP among WLHIV in Busia County, Kenya, the findings revealed critical insights into contraceptive variety and stock status. The analysis indicated a general commitment to offering a variety of contraceptive options to WLHIV in Busia County, Kenya. However, the variety of contraceptives available was affected by stock status, training gaps and competency of staff for provision of permanent methods, and funding limitations, impacting the ability to provide a comprehensive range of PPFP options consistently, this highlighted challenges in providing a variety of contraceptives due to stock-outs.

*“Someone will come and she will say that she wants long term maybe for three years that one is out of stock.*.. *One year.” -****KII-13***

*“Okay there suppliers that sometimeswe can have a few out of stock… like DEPO*… *so we rarely miss completelywe can miss 1 or 2but then they are supplied again. –****KII- 4***

*“Again we used to have uh Marie stopes supporting us with BTL*,* but they*, *since they left*,* none.-****KII-1***

*“No*,* the BTLs are usually done by Maria Stopes.We’ve not had anybody doing a BTL” –****KII-2***

*“Mostly*,* it’s the government supply… sometimes partners chip in*, *but it’s not always enough.“****- KII-BA-Resp 11***

*“We face regular stock-outs*, *especially for long-term methods… it’s a continuous struggle to keep up.“****- KII-BW-Resp 15***

## Discussion

The present study stands out as one of a few studies to examine consistent modern PPFP uptake among WLHIV in Kenya. Most published studies have examined PPFP use among the general population of postpartum women and/or focused primarily on the current use of PPFP. The study findings revealed that only one in two WLHIV utilized PPFP consistently within the first year postpartum. A study in Uganda examining PPFP uptake among WLHIV reported a lower uptake rate of 40% [18], however, it reported current PPFP use while the present study reports on consistent use through the first year postpartum. At Webuye Hospital, western Kenya, a higher current PPFP uptake at 78% was reported but not specifically among WLHIV [19]. However, this higher rate aligns with the ever-use rate of PPFP observed by the present study (73.25%). Findings based on Kenya DHS data reported a 50% PPFP use among the general population of postpartum women, a finding comparable to the consitent PPFP use rate among PLHIV observed by the present study [20]. Consequently, the present study study findings point towards a low consistent use of PPFP and a high drop out of PPFP use among WLHIV (73.25% ever used PPFP versus 52.55% who consistent used PPFP) which could be one of the missing links in narrowing the MTCT rate in Kenya [10].

The study findings also showed that the perception of being adequately informed about all available PPFP methods increased the likelihood of using PPFP consistently. Comparable in low income countries, studies have shown that providing adequate information on FP improves the uptake rates [21, 22]. This finding underscores the importance of effective communication and education within the healthcare system regarding contraceptive options [23]. When WLHIV feel knowledgeable about the range of PPFP methods, including their benefits and suitability, they are more likely to make informed decisions aligning with their reproductive health goals [24].

The study findings also revealed significant concerns about staffing levels across healthcare facilities. Respondents emphasized the inadequacy of the workforce to meet the demand for PPFP services among other services. Staff shortages remain a persistent challenge in the African healthcare system with SSA where Kenya lies one of the most affected regions globally [25]. This leads to longer wait times, limits access to comprehensive services, overwhelms health workers and compromises the quality of care [26]. This situation is exacerbated by a disproportionately high burden of HIV in the county compared to other areas in Kenya [10]. As a result, this circumstance could have contributed to the low consistent uptake of PPFP among WLHIV and the high dropout rates observed in the present study.

Moreover, the study findings revealed gaps in staff training, particularly in newer or specific PPFP guidelines, posing challenges to healthcare workers in delivering up-to-date and comprehensive care. Quotes highlighted the lack and need for continuous professional development in PPFP guidelines and practices. Similar sentiments were also expressed by health workers in a study conducted in South Africa, Uganda, and Kenya [27]. Therefore, the study findings demonstrate a possible persistent challenge related to the adequate training of health workers, an important aspect that a study in Narok County, Kenya, found critical for improving staff motivation [28]. Without adequate training, healthcare providers may lack the necessary knowledge and skills to offer effective PPFP services, potentially leading to suboptimal care outcomes [29].

Intrinsic motivation, personal commitment, and the desire to achieve targets were evident among staff. Similar observations have been noted by several researchers, underscoring the importance of fostering intrinsic motivation among healthcare workers to enhance service delivery [30–32]. While these factors may have contributed to the level of consistent PPFF uptake among WLHIV observed by the study, external factors such as workload and resource constraints which were also highlighted by the health workers could also have influenced motivation levels. Additionally, the lack of training may have been a contributing factor affecting motivation levels [28]. The issue of low motivation among health facility staff was highlighted in a study conducted in Pakistan as one of the key drivers of low FP uptake which could also in part explain the findings of the present study [33].

The study identified a lack of regular, structured support supervision visits by senior health officials to healthcare facilities in Busia County, Kenya. Support supervision is a key aspect of improving the quality of care [34]. Studies have shown that health workers perceived an improvement in their performance and attributed this to the supportive supervision [35]. This deficiency hinders the ability of healthcare workers to receive on-site guidance, review service delivery practices, and address challenges affecting PPFP service provision effectively. The absence of such supervision can result in inefficiencies and gaps in PPFP service delivery, ultimately impacting the quality of care received by WLHIV.

Health information use is a key aspect of the WHO Health Systems Building Blocks framework [17]. The study identified variations in the use and management of health information within healthcare facilities in Busia County, Kenya, particularly regarding PPFP services for WLHIV. While some facilities attempted regular data reviews to improve service delivery, specific data targeting consistent PPFP uptake among WLHIV was not consistently analyzed or utilized for decision-making. Quotes highlighted the lack of distinct review or utilization of PPFP data for WLHIV, indicating a gap in strategic data management practices. Similar studies have found that overall, data use at the primary service provision centres is generally low thereby hindering data use [36, 37]. The findings suggest that healthcare facilities may not be fully utilizing available data to identify trends, monitor progress, and inform targeted interventions to improve PPFP service delivery to WLHIV.

The study findings also revealed both efforts and obstacles in providing a diverse range of contraceptive options to WLHIV in Busia County, Kenya. While there was a general commitment to offering various contraceptives, challenges such as stock status and stockouts hindered the consistent provision of a comprehensive range of PPFP options or any PPFP method at all. Previous studies have shown that contraceptive use improves with the availability of more contraceptive options [38, 39]. In the present study, there were instances where certain contraceptives, particularly long-term methods like 3 months implants, Bilateral Tuba ligation (BTL), were unavailable due to stock-outs, leading to referrals to gynecologists respectively. This situation indicated a gap in the availability of choice options for WLHIV seeking PPFP services, limiting their ability to select a preferred method. This limitation could have resulted in lower uptake or consistency of use [40]. Consequently, it underscores the need for improved supply chain management and staff training to ensure the consistent availability and provision of a wide array of contraceptives.

This study, while aiming to shed light on the health system factors influencing the uptake of PPFP among WLHIV in Busia County, Kenya, possessed certain limitations. Firstly, the reliance on retrospective data restricted our ability to establish causal relationships between health system factors and PPFP uptake. Secondly, potential sampling bias arose from the selection of WLHIV accessing ART services in health facilities, excluding those not in active care or seeking services from alternative sources. Thirdly, self-reported data from the study might have been susceptible to recall and social desirability biases [41]. Moreover, the study was confined to health facilities in Busia County, Kenya and among health-service seeking WLHIV who are most likely to utilize various aspects of health services. Some community-based studies have found a much lower uptake PPFP uptake [42]. However, the study used both quantitative data collection and qualitative insights, which gave a more nuanced understanding of their experiences and perspectives regarding PPFP and health system factors. Moreover, the study triangulated data reported with health facility records to establish the accuracy of the information, in addition to training research assistants on probing and the use of verification questions. This ensured that the study findings were as reliable as reasonably possible.

## Conclusions and implications

The study found that the uptake of PP modern FP methods was at 70% among WLHIV within the first year postpartum though only half consistently used the methods within the first 12 months postpartum. Secondly, the study found that being well-informed about all the PP modern FP methods options significantly enhances utilization among WLHIV. This highlights the necessity for effective communication and education within healthcare settings to improve awareness and understanding of available PP modern FP methods, thereby supporting informed decision-making. Thirdly, the study found that staff shortages and insufficient training on PPFP guidelines were major concerns. These challenges led to longer wait times, decreased access, and potentially lower quality of care, affecting PP modern FP methods uptake. Despite these obstacles, intrinsic motivation among healthcare workers played a critical role in service delivery, though it was also influenced by external pressures and the need for more comprehensive training and support. Lack of regular support supervision and suboptimal use of health information for PPFP services were identified as barriers, indicating a need for improved management practices and data utilization to enhance service delivery and decision-making. Finally, the study found that the availability and diversity of contraceptive options were crucial for PP modern FP method uptake. However, challenges such as stockouts hindered the provision of comprehensive PPFP services. This emphasized the need for better supply chain management and training to ensure a wide range of contraceptive methods are available to meet the preferences of WLHIV.

## Electronic supplementary material

Below is the link to the electronic supplementary material.

Supplementary Material 1

## Data Availability

To improve data transparency, a de-identified dataset is included with this submission. For additional information, please contact tebbyflorah@gmail.com.
